# Alteration of CYP4A11 expression in renal cell carcinoma: diagnostic and prognostic implications

**DOI:** 10.7150/jca.36438

**Published:** 2020-01-14

**Authors:** Sup Kim, Jin Man Kim, Hyo Jin Lee, Jae Sung Lim, In-Ock Seong, Kyung-Hee Kim

**Affiliations:** 1 Department of Radiation Oncology, Chungnam National University Hospital, Daejeon, South Korea;; 2 Department of Pathology/Medical Science, Chungnam National University School of Medicine, Daejeon, South Korea;; 3 Division of Hematology/Oncology, Department of Internal Medicine, Chungnam National University School of Medicine, Daejeon, South Korea;; 4 Department of Urology, Chungnam National University School of Medicine, Daejeon, South Korea.

**Keywords:** cytochrome P450 CYP4A11, peroxisome proliferator-activated receptor-α, renal cell carcinoma.

## Abstract

**Background**: Cytochrome P-450 4A11 (CYP4A11) and peroxisome proliferator-activated receptor-α (PPARα) are expressed at high levels in renal proximal tubules, and upregulation of CYP4A11 protein levels is known to be influenced by PPAR agonists. The goal of this study was to evaluate the clinicopathological role of CYP4A11 expression in renal cell carcinoma (RCC).

**Methods**: We performed immunohistochemical analysis of CYP4A11, CYP4A22 and PPARα and correlated the results with the clinicopathological features of RCC (n=139). Reverse transcription digital droplet polymerase chain reaction (RT-ddPCR) against CYP4A11 and CYP4A22 was also performed.

**Results**: CYP4A11 mRNA expression levels were higher in non-neoplastic kidney tissues than in matched tumor tissues in 12 matched pairs of freshly frozen primary clear-cell RCC (ccRCC) and nontumor tissue (p=0.002). Immunohistochemical staining showed that CYP4A11 expression was significantly lower in ccRCC than in non-ccRCCs, including papillary, chromophobe, and unclassified RCCs (p<0.001). CYP4A11 expression was associated with PPARα expression, males and high nuclear histologic grades (p=0.001, p=0.018 and p<0.001). Univariate and multivariate analyses revealed that CYP4A11 expression was correlated with short overall survival (p=0.007 and p=0.010).

**Conclusion**: These findings suggest that CYP4A11 expression is a potential poor prognostic factor of RCC. The considerable decrease in CYP4A11 expression is a predictive diagnostic factor of ccRCC, and CYP4A11 metabolism in ccRCC might be different from that in non-ccRCCs.

## Introduction

Renal cell carcinoma (RCC) is a group of different types of cancer arising from the renal epithelium 1. The three major types of RCC are clear-cell RCC (ccRCC), papillary RCC (pRCC), and chromophobe RCC (chRCC), of which ccRCC is most common 2. Each RCC subtype is characterized by a cancer-specific mutational spectrum that is often linked to different metabolic pathways involved in oxygen, iron, energy and/or nutrient sensing 2-4. RCC cells can process different metabolic features from normal tubular epithelial cells and use this metabolic conversion to overcome stress imposed on the tumor cells. Understanding each tumor-specific process can lead to improved diagnosis and prognosis and to the development of novel therapeutics.

Physiologically, members of the cytochrome P-450 4 (CYP4) family catalyze the omega (ω) hydroxylation of saturated, branched-chain, and unsaturated fatty acids 5. In addition to a playing a in fatty acid catabolism, the CYP4 family also catalyzes the formation of the ω-hydroxylated metabolite of arachidonic acid, 20-hydroxyeicosatetraenoic acid (20-HETE), which is a vasoactive and natriuretic substance that regulates vascular and renal functions 6. The human CYP4A subfamily consists of two highly homologous CYP4A genes, namely, CYP4A11 and CYP4A22. CYP4A22 is known to be a nonfunctional enzyme and is expressed at much lower levels than CYP4A11 5. CYP4A11 harbors the peroxisome proliferator-activated receptor-α (PPARα) response element in the promoter region of the gene; therefore, PPARα can regulate CYP4A11 7. Both CYP4A11 and PPARα were expressed in the renal proximal tubular epithelium 8, and the PPARα agonist clofibrate induced CYP4A protein expression and activity in the renal cortex 8.

The aim of the study was to determine the cellular localization and immunoreactivity levels of CYP4A11, CYP4A22 and PPARα by immunohistochemistry (IHC) in 108 ccRCCs and 31 non-ccRCCs. Additionally, western blotting and reverse transcription digital droplet polymerase chain reaction (RT-ddPCR) were performed. The results of the IHC study were correlated with various clinicopathological characteristics, including patient survival.

## Materials and Methods

### Patients and tissue samples

This study was approved by the Institutional Review Board of Chungnam National University Hospital (CNUH 2018-02-017-003). All tissue samples for western blot and RT-PCR studies using frozen tissue samples and clinical data were obtained from the National Biobank of Korea at Chungnam National University Hospital. All patients signed a written informed consent form for biobanking before data were included in the register. The requirement for informed consent for the retrospective comparison study was waived because the study was based on immunohistochemical analysis using formalin-fixed paraffin-embedded (FFPE) tissue.

We conducted a review of the records of 214 patients who underwent surgical resection of RCC between 1999 and 2014 at Chungnam National University Hospital in Daejeon, South Korea. The inclusion criteria were that the FFPE tumor tissues were available and the follow-up data were detailed. The exclusion criteria were as follows: (1) patients had previous history of other cancers; (2) patients had received previous curative resection for any kidney lesion; (3) patients had received preoperative chemotheraphy or radiation therapy; (4) patients had received any molecular targeted therapy. After applying both inclusions and exclusion criteria, 139 patients with RCC were included in the study. The 139 RCC cases included 108 cases of ccRCC, 18 cases of type 2 pRCC, 4 cases of chRCC and 9 cases of unclassified RCC. All electronic medical records of the patients were reviewed by KHK and HJL to obtain clinical data. In one case, there was a regional lymph node metastasis in a category-3 primary tumor, and the other 138 cases had no regional lymph node metastasis or distant metastasis at the time of the initial surgical resection. Eighty-seven patients among the 139 RCC cases underwent immunotherapy. The type of immunotherapy provided was interferon therapy alone, without a checkpoint inhibitor. RCC recurrence or metastasis was determined via imaging and/or histological analysis. Disease-free survival (DFS) was determined as the time interval between the date of initial surgical resection and the date of RCC recurrence or metastasis. Overall survival (OS) was defined from the time of initial surgical resection to the date of death due to any cause. Without confirmation of death, recurrence or metastasis, OS or DFS time was recorded based on the last known date that the patient was alive. The 2 most representative viable tumor areas and one non-neoplastic area were selected and marked on the hematoxylin and eosin (H&E)-stained slides. The tumor, node, and metastasis (TNM) staging and nuclear histologic grading for RCC were performed based on the time of surgical resection according to the staging system of the 8^th^ Edition of the American Joint Committee on Cancer (AJCC) 9. Tissue microarrays (TMAs) were created by punching tissue columns (3.0 mm in diameter) from the original paraffin blocks and inserting the columns into new recipient paraffin blocks (each containing 30 holes to receive the tissue columns). Four and 12 matched pairs of freshly frozen primary ccRCC and non-neoplastic kidney tissue were obtained for western blotting and RT-PCR, respectively, from the National Biobank of Korea at Chungnam National University Hospital, a member of the Korean Biobank Network.

### Immunohistochemical staining analysis

Immunohistochemical staining of the tissue sections from the TMA paraffin blocks was performed by Discovery UltraMap-HRP detection and ChromoMap DAB detection using a Ventana Discovery XT automated immunostainer (Ventana Medical Systems Inc., Tucson, Arizona). A primary rabbit polyclonal antibody against human CYP4A11 (product # PA5-30004, diluted 1:100; ThermoFisher Scientific, Rockford, IL, USA), a primary rabbit polyclonal antibody against human CYP4A22 (product # PA5-30004, diluted 1:100; ThermoFisher Scientific, Rockford, IL, USA) and a mouse monoclonal antibody against human PPARα (product # MAB12349, diluted 1:100; Abnova, Taipei City, Taiwan) were used (incubation at 31 °C, 32 min).

Immunohistochemical staining was scored using digitally scanned files and the ScanScope program (Aperio ScanScope CS system, Vista, CA, USA). A modified version of the method described by Allred et al. was used to evaluate both the intensity of immunohistochemical staining and the proportion of stained neoplastic or non-neoplastic hepatocytes in each slide 10. The proportion scores ranged from 0 to 5 (0, 0; 1, >0 to 1/100; 2, >1/100 to 1/10; 3, >1/10 to 1/3; 4, >1/3 to 2/3; 5, >2/3 to 1), and the intensity scores ranged from 0 to 3 (0, negative; 1, weak; 2, moderate; and 3, strong). To determine the total immunohistochemical score, the intensity score and the proportion score were multiplied for each specimen (range, 0-15). For categorical analyses, expression at greater than the median value of the total score of CYP4A11 was regarded as high (total score>4). The results were examined separately and scored by KHK and JMK, who were blinded to patient details. Discrepancies in the scores were discussed to obtain a consensus.

### Western blot assay

Samples from 4 ccRCC patients, 4 paired vials (100 mg) of ccRCC tumor tissue and one nontumor tissue, at a distance of at least 2.0 cm from the tumor, were stored at -80°C in liquid nitrogen and subsequently examined for CYP4A and CYP4A11 expression by western blotting. A primary rabbit monoclonal antibody against human CYP4A (homologous to human CYP4A11 and 4A22; ab140635; Abcam, Cambridge, UK) and a primary rabbit polyclonal antibody against human CYP4A11 (product # PA5-30004; ThermoFisher Scientific, Rockford, IL, USA) were diluted 1:1000. Western blotting was conducted as previously described 11.

### Reverse transcriptase digital droplet PCR (RT-ddPCR)

The 12 paired ccRCC tissue and nontumor tissue sections were stored at -80°C in liquid nitrogen and subsequently examined for CYP4A11 and CYP4A22 mRNA expression by RT-ddPCR. Total RNA was isolated with TRIzol reagent (Thermo Fisher Scientific, Waltham, MA, USA) in accordance with the manufacturer's instructions. The same quantity of total RNA was reverse transcribed to complementary DNA (cDNA) using a cDNA synthesis master mix (ReverTra Ace® qPCR RT Master Mix with gDNA Remover, Toyobo Co., Ltd., Osaka, Japan). The following primers were prepared: CYP4A11 forward primer sequence: 5'-CTCAACACAGCCACGCTTTC-3́ and reverse primer sequence: 5'-ACAAGTCGTGCAATGGGGAT-3' (input PCR template: NM_001319155.1) and CYP4A22 forward primer sequence: 5'-TGGCCCAACCTAGAGGTGTT-3́ and reverse primer sequence: 5'-AGGACGTCTCACCTTGATCCT-3́ (input PCR template: NM_001308102.1).

The QX200™ Droplet Digital™ PCR system (Bio-Rad) was used for RT-ddPCR against CYP4A11 and CYP4A22. ddPCR was conducted as previously described 12. The 20-μL PCR mix contained QX200™ ddPCR™ EvaGreen Supermix (Bio-Rad), 300 nM each primer and approximately 50 ng of cDNA template.

### Statistical analyses

The relationships between CYP4A11 expression and the clinicopathological parameters were evaluated using Pearson's chi-square test and the Mann-Whitney U test. Differences in CYP4A11 mRNA expression between the paired RCC tissue and nontumor tissue sections were assessed using the Wilcoxon signed-rank test. Postoperative OS and DFS were determined using Kaplan-Meier survival curves and a log-rank test. The Cox proportional hazards model was applied for univariate and multivariate survival analyses. Statistical significance was set at p<0.05 (SPSS v.24; SPSS Inc., Chicago, IL, USA).

## Results

### Association of clinicopathological characteristics with expression of CYP4A11, CYP4A22 and PPARα

**Figure 1:**
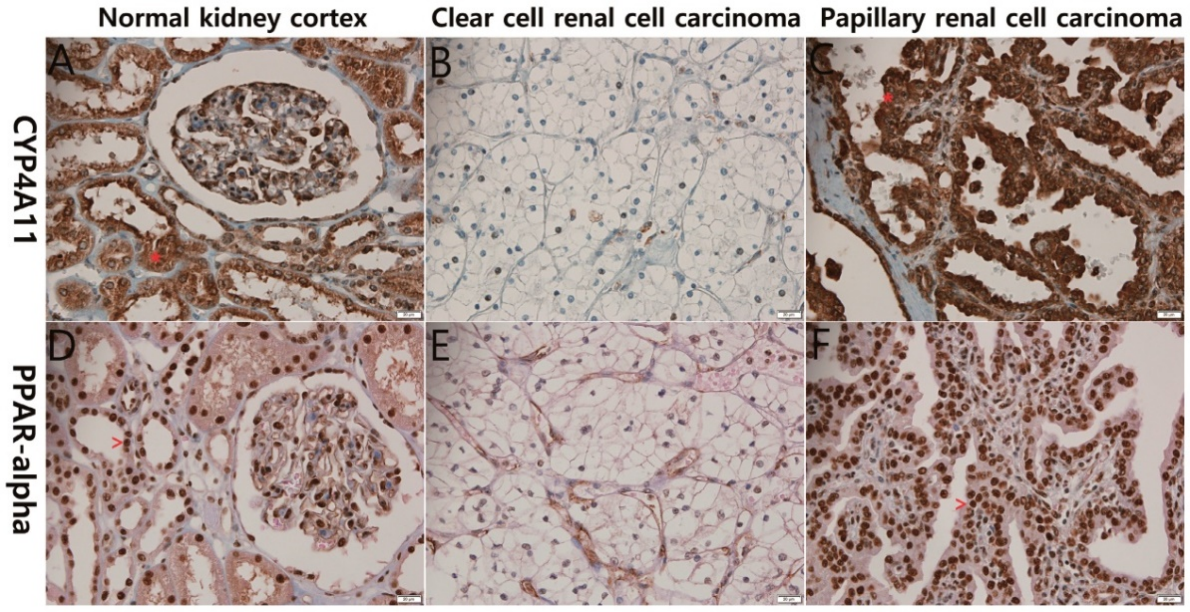
Representative photographs of CYP4A11 and PPARα immunohistochemical staining of the normal kidney cortex, clear-cell renal cell carcinoma (ccRCC) and papillary renal cell carcinoma. The normal renal tubular epithelial cells (A and D) and papillary renal cell carcinoma cells (C and F) show strong positive cytoplasmic staining for CYP4A11 (*) and positive nuclear staining for PPARα (^), in contrast to the ccRCC cells (B and E), which exhibit weak staining (scale bar = 20 μm).

The 139 RCC cases were evaluated immunohistochemically for CYP4A11 expression in RCC tissues. Almost all of the non-neoplastic proximal tubules were strongly and diffusely positive for CYP4A11 and PPARα expression, while most of the ccRCC cells showed severely decreased expression of CYP4A11 and PPARα. The non-ccRCC cells, including those of the papillary type 2, chromophobe and unclassified types, expressed higher levels of CYP4A11 and PPARα than ccRCC cells (p<0.001 and p<0.090) (Fig. 1). Most non-neoplastic proximal tubules were weakly and diffusely positive for CYP4A22 expression, but 13 (9.3%) of the 139 RCCs were weakly expressed.

**Figure 2:**
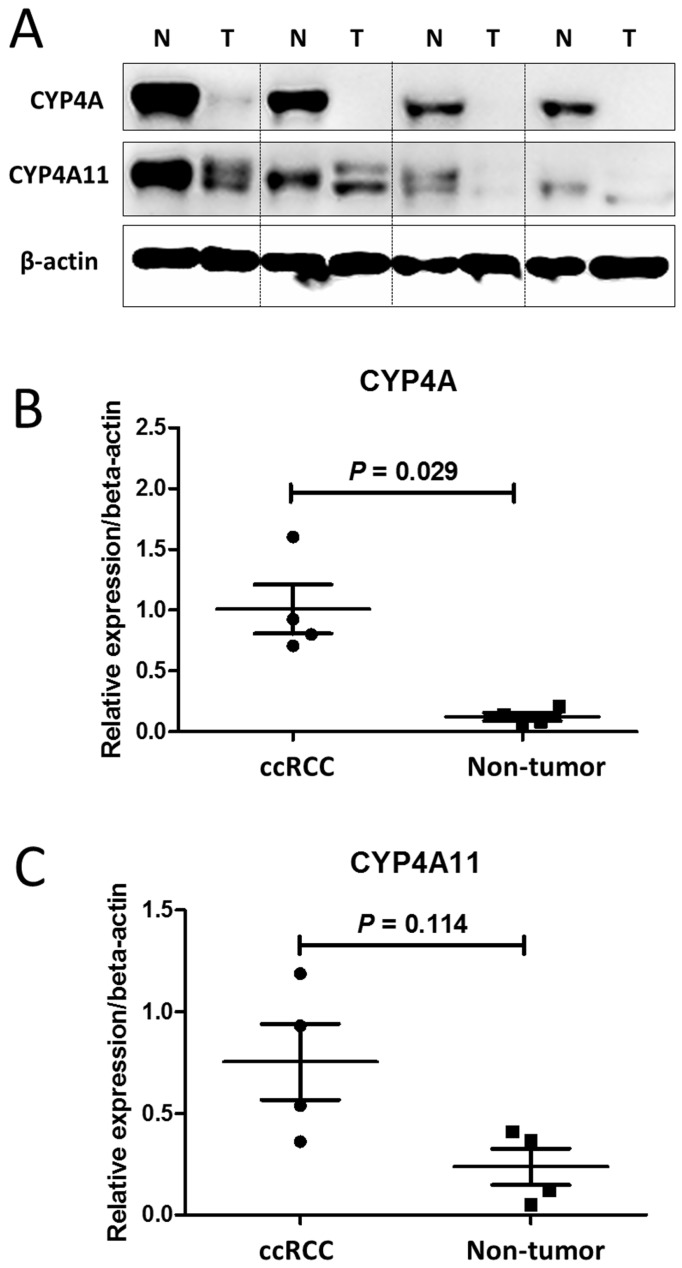
Western blot analysis of CYP4A and CYP4A11 in 4 matched pairs of clear-cell renal cell carcinoma (ccRCC) tissue and nontumor tissue sections. The ccRCC tumor tissue samples expressed significantly lower levels of CYP4A and CYP4A11 than the nontumor tissue samples. (A) Cell lysates were collected and subjected to western blot analysis for CYP4A and CYP4A11. (B) Relative intensity of CYP4A protein expression in the ccRCC tumor and nontumor tissue sections (P = 0.029; Wilcoxon signed-rank test). (C) Relative intensity of CYP4A11 protein expression in the ccRCC tumor and nontumor tissue sections (P = 0.114; Wilcoxon signed-rank test).

Regarding the immunohistochemical staining, western blot assays of CYP4A11 expression in the 4 matched pairs of ccRCC and nontumor tissue sections showed that ccRCC tumor samples expressed significantly lower levels of CYP4A (homologous to human CYP4A11 and 4A22) and CYP4A11 than the non-neoplastic tissue samples (p=0.029, p=0.114) (Fig. 2).

The clinicopathological characteristics of the 139 RCC patients associated with CYP4A11 expression are presented in Table 1. High CYP4A11 expression in the 139 RCCs was positively associated with PPARα expression, males, the non-ccRCC type, and high histologic grades (grade 1/2 versus grade 3/4) (p=0.001, p=0.018, p<0.001 and p<0.001).

**Figure 3:**
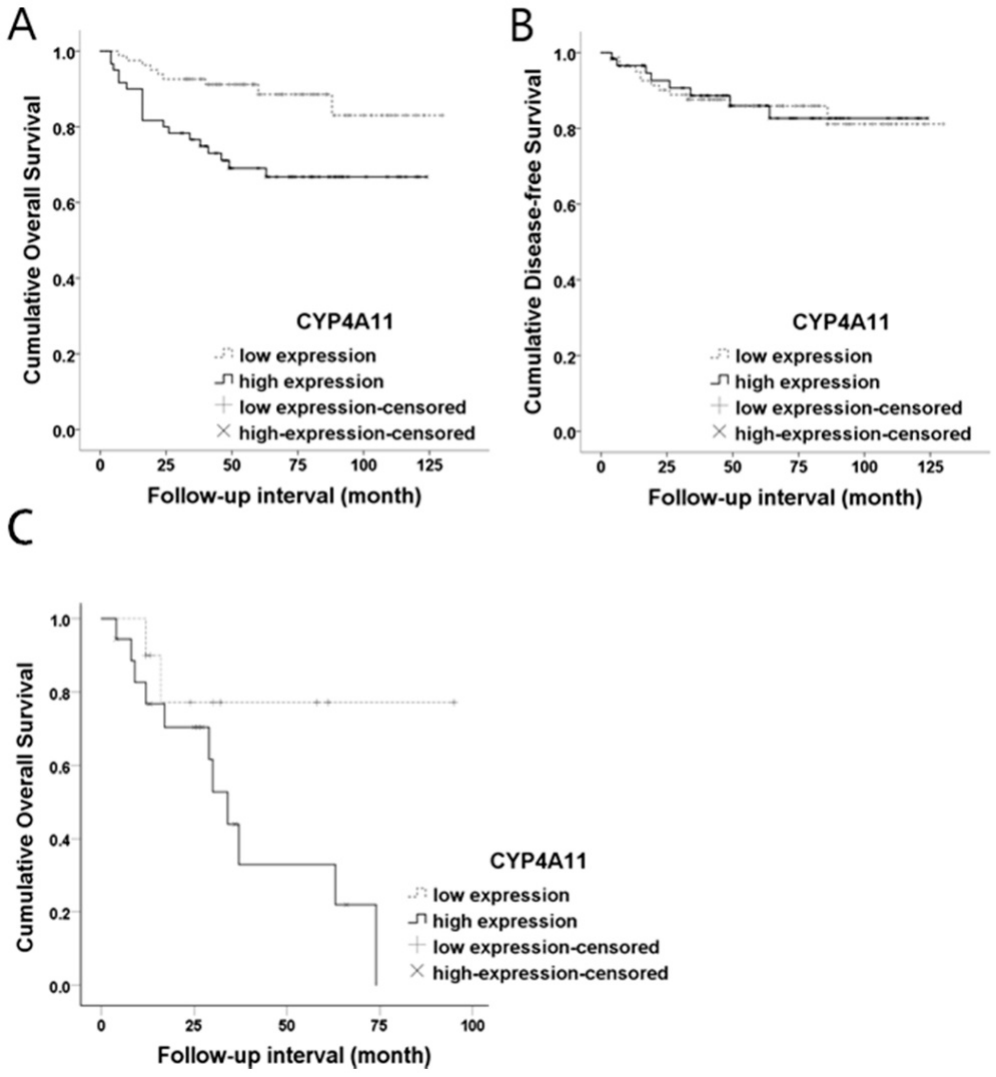
Kaplan-Meier overall survival curves for CYP4A11 protein expression levels in renal cell carcinoma. **(A)** High CYP4A11 expression was associated with short overall survival (p=0.005; log-rank test). **(B)** The CYP4A11 expression levels did not show statistical significance in terms of the disease-free survival outcome (p=0.945; log-rank test). **(C)** The CYP4A11 mRNA expression levels are marginally significant prognostic factors for overall survival in GSE2748 (p=0.08; log-rank test).

Both OS and DFS analyses were performed for the 139 RCC patients. Kaplan-Meier survival curves and log-rank tests showed a significant association between high CYP4A11 expression and short OS (log-rank=7.994, p=0.005), while no association with DFS was observed (log-rank=0.005, p=0.945) (Fig. 3). In the univariate analysis, CYP4A11, old age, the non-ccRCC type, high histologic nuclear grade, and high pathologic stage were significantly associated with short OS (Table 2). Multivariate analyses using Cox's proportional hazard regression model were performed for CYP4A11 expression, PPARα expression, age, sex, and pathologic stage. In the multivariate analysis, increased CYP4A11 expression and high pathologic stage were independent poor prognostic factors indicating short OS (p=0.010 and p=0.023, respectively) (Table 3). To support our data in which increased CYP4A11 expression of RCC cells positively correlated with shorter OS, we downloaded GSE2748 entitled “A molecular classification of papillary renal cell carcinoma” (https://www.ncbi.nlm.nih.gov/geo/query/acc.cgi?acc=GSE2748) and analyzed the correlation between CYP4A11 mRNA expression and OS period. Of the 34 patients with pRCC, clinical data of 19 patients with pRCC were available in GSEA2748. The OS periods tended to have shorter periods in pRCC patients with a high CYP4A11 expression group than a low CYP4A11 expression group (p=0.08) (Fig. 3).

CYP4A22 and PPARα expression did not show an association with the clinicopathological characteristics of the 139 RCC patients.

### CYP4A11 and CYP4A22 mRNA expression levels in 12 matched pairs of ccRCC and non-neoplastic tissues

**Figure 4:**
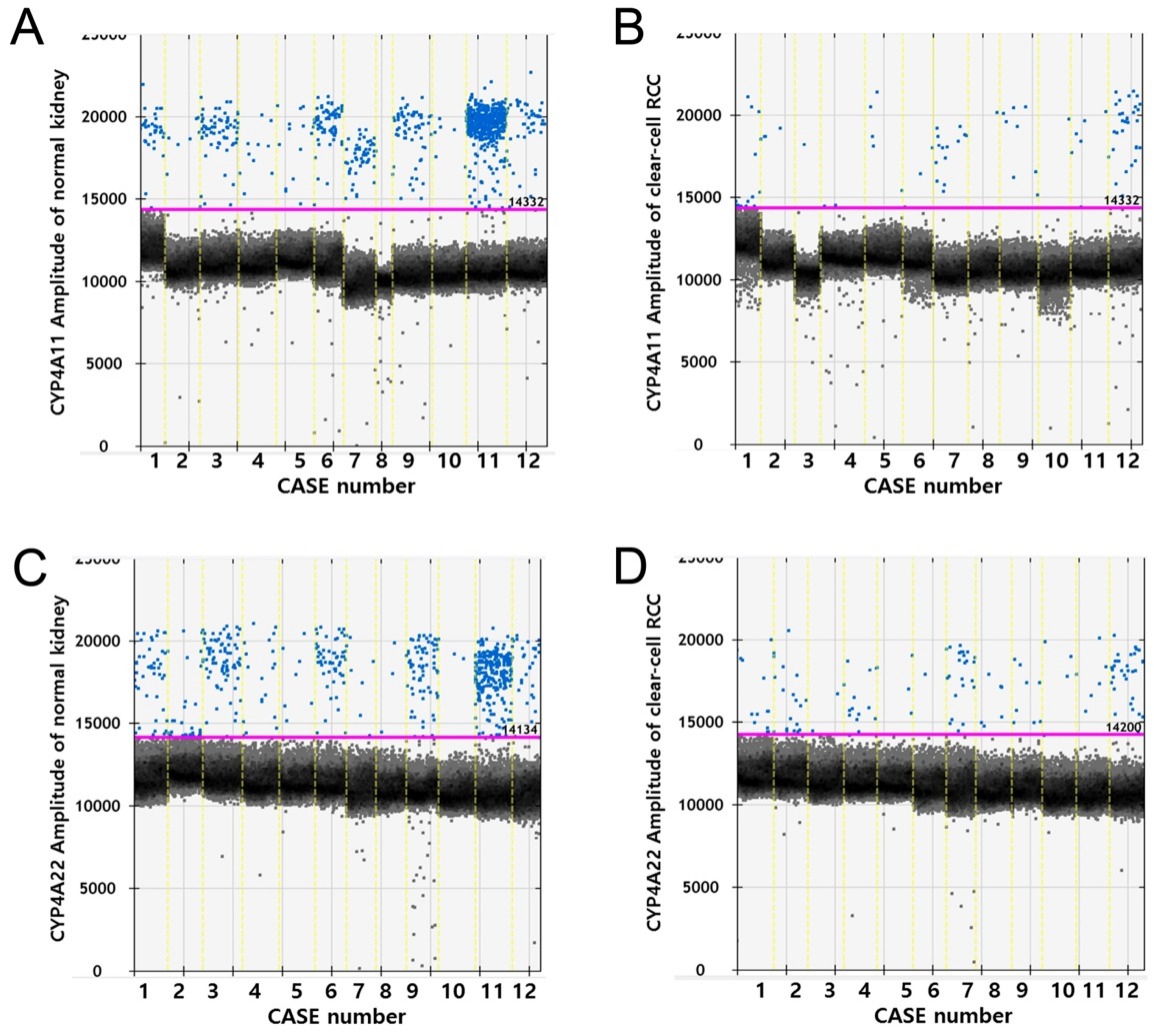
Droplet digital PCR amplification plots for individual cases. Comparison of CYP4A11 and CYP4A22 mRNA expression levels by reverse transcriptase digital droplet PCR (RT-ddPCR) in 12 paired clear-cell renal cell carcinoma and nontumor tissue samples. Positive droplets are shown in blue, and negative droplets are shown in black/gray. The pink line represents the threshold, dichotomizing positive and negative droplets. (p = 0.002 and p = 0.012, Wilcoxon signed-rank test).

RT-ddPCR analysis of CYP4A11 and CYP4A22 mRNA in 12 matched pairs of ccRCC tissue and non-neoplastic renal cortical tissue from 12 patients showed higher copy numbers of CYP4A11 and CYP4A22 in non-neoplastic tissue than in ccRCC tissue (p=0.002 and p=0.012, Wilcoxon signed-rank test). The average number of copies of CYP4A11 and CYP4A22 in the 50-ng cDNA template in the ccRCC tissue/non-neoplastic renal cortical tissue was 14.6/171.3 and 22.4/123.0, respectively (Fig. 4).

## Discussion

In this study, we evaluated the expressions of CYP4A11, CYP4A22 and PPARα in 139 RCC cases. We demonstrated that the CYP4A11 expression was significantly lower in ccRCC cells than in non-ccRCC. In addition, increased CYP4A11 protein and mRNA expression in RCC cells was positive correlation with a shorter OS period and could be considered one of the potential poor prognostic factors. The different expressions of CYP4A11 between ccRCC and non-ccRCC can be correlated with different metabolism of each cancer type.

RCC cells can process nutrient molecules differently from normal tubular epithelial cells. This unique metabolic process is controlled by specific genetic mutations that are associated with cell growth advantage 2. Understanding the unique metabolic pathways of RCCs provides an effective approach to diagnosis and treatment.

RCC has a variety of subtypes with varying histological and clinical outcomes due to the different metabolism of each subtype 13. The classic subtype of RCC is ccRCC, and the other major subtypes are pRCC, chRCC and undifferentiated RCC 2, 14. The most common gene involved in the pathogenesis of ccRCC is the von Hippel-Lindau gene (VHL) 14. One of the major functions of the VHL gene product is regulation of the levels of hypoxia-inducible factor 1 alpha and 2 alpha (HIF1A and HIF2A) 15. In ccRCC, inactivation or loss of VHL leads to aberrant accumulation of HIF proteins, which in turn results in angiogenesis, glycolysis, apoptosis, and lipid deposition in ccRCC 2, 16. Accordingly, in terms of histological appearance, the typical ccRCC is rich in glycogen, lipids and blood vessels 14. We hypothesized that the level of CYP4A11 in ccRCC cells could be related to the lipid- rich cytoplasm of ccRCC cells. CYP4A is well known to catalyze the oxidation of endogenous lipids and xenobiotics 17. CYP4A shows a preference for the metabolism of medium-chain fatty acids in lipid homeostasis 18. According to previous studies, CYP4A mRNA expression is upregulated in human thyroid, ovary, breast, and colon cancer tissues and in pancreatic ductal adenocarcinoma tissues 19-21. However, CYP4A11 was downregulated in hepatocellular carcinoma 11, although the liver and kidney showed the highest levels of CYP4A11 mRNA expression 22.

PPARs regulate the expression of genes that control fatty acid metabolism and bind to peroxisome proliferator response elements in CYP4A 7. In particular, CYP4A11 is regulated by PPARα and is expressed at high levels in the liver and kidney 5, 8, 23, 24. Our data also showed that the expression of CYP4A11 and PPARα was positively related to each other in RCC cases. CYP4A11 can catalyze the ω-hydroxylated metabolite of arachidonic acid, 20-HETE, which plays an important role in the regulation of vascular tone, renal blood flow, and renal tubular sodium transport 8, 25. Each RCC type exhibits unique cancer metabolism; ccRCC cells contain more total cholesterol, especially esterified cholesterol, than normal renal tubular epithelial cells or non-ccRCC cells 2, 26. Those results of lipid deposition in ccRCC cells are consistent with our data of decreased CYP4A11 expression in ccRCC cells. Therefore, we suggest that decreased CYP4A11 expression could be a suitable diagnostic marker for ccRCC. Reduced expression of CYP4A11 may be one of the characteristics of ccRCC that is different from normal renal tubular epithelial cells and may be one of the causes of decreased fatty acid catabolism or increased lipogenic metabolism of ccRCC. The genetic or molecular biological difference between each tumor type underlies different morphological features 27.

The present study is the first to evaluate the expression level of the human CYP4A11 protein and mRNA in human RCC. CYP4A11 expression was reduced in ccRCC cells compared to non-neoplastic renal tubular epithelial cells or non-ccRCC cells based on IHC, western blotting and RT-ddPCR. In the 139 RCCs, CYP4A11 expression was positively correlated with poor prognostic factors, including high histologic nuclear grade and short OS. Our results demonstrate that CYP4A11 metabolism is different between ccRCC and non-ccRCCs and might be useful as a predictive diagnostic factor for ccRCC and for the development of a potential therapeutic target.

## Figures and Tables

**Table 1:** Patient characteristics according to immunohistochemical expression of CYP4A11 in renal cell carcinoma (n=139).

Characteristics	Total no. (%)	CYP4A11 expression		
		Low (%)	High (%)	P
		79 (100.0)	60 (100.0)	
PPARα expression (median (IQR))	139 (100.0)	4 (2.75-8)	6 (4-10)	0.001*
Age (y) (median (IQR))	139 (100.0)	66 (55-71)	65 (53-72)	0.789*
Tumor size (median (IQR))	139 (100.0)	5.0 (3.2-6.3)	5.5 (4.0-8.0)	0.328*
				
Gender				0.018**
Female	40 (28.8)	29 (36.7)	11 (18.3)	
Male	99 (71.2)	50 (63.3)	49 (81.7)	
Histologic type				<0.001**,^†^
Clear cell	108 (77.7)	75 (94.9)	33 (55.0)	
Papillary type 2	18 (12.9)	3 (3.8)	15 (25.0)	
Chromophobe	4 (2.9)	1 (1.3)	3 (5.0)	
Unclassified	9 (6.5)	0 (0.0)	9 (15.0)	
Histologic nuclear grade				<0.001**,^‡^
I	0 (0.0)	0 (0.0)	0 (0.0)	
II	74 (53.2)	58 (73.4)	16 (26.7)	
III	47 (33.8)	21 (26.6)	26 (43.3)	
IV	18 (12.9)	0 (0.0)	18 (30.0)	
Pathologic stage				0.773**,^‡^
I	61 (43.9)	36 (45.6)	25 (41.7)	
II	22 (15.8)	12 (15.2)	10 (16.7)	
III	55 (39.6)	31 (39.2)	24 (40.0)	
IV	1 (0.7)	0 (0.0)	1 (1.7)	

*, Mann-Whitney U test; IQR, interquartile range; **, Pearson's chi-square tests; ^†^, clear-cell renal cell carcinoma versus others; ^‡^, I-II versus II-IV.

**Table 2:** Results of univariate analysis of overall survival and disease-free survival in 139 patients with renal cell carcinoma.

Prognostic factor	Overall survival		Disease-free survival	
	HR (95% CI)	P*	HR (95% CI)	P*
CYP4A11 expression	1.113 (1.029-1.204)	0.007	1.021 (0.914-1.140)	0.716
PPARα expression	0.982 (0.890-1.082)	0.708	0.923 (0.810-1.051)	0.225
Age at operation	1.043 (1.008-1.080)	0.015	1.006 (0.969-1.044)	0.764
Sex				
Female	1 (reference)		1 (reference)	
Male	2.712 (0.940-7.818)	0.065	1.672 (0.555-5.041)	0.361
Histologic type				
Clear-cell type	1 (reference)		1 (reference)	
Non-clear-cell types	3.385 (1.609-7.123)	0.001	1.416 (0.509-3.941)	0.506
Histologic nuclear grade		<0.001		0.641
I	1 (reference)		1 (reference)	
II	2.858 (1.057-7.732)	0.039	1.227 (0.457-3.296)	0.684
III	10.402 (3.838-28.197)	<0.001	1.871 (0.505-6.926)	0.348
IV	NA	NA	NA	NA
Pathologic stage		0.004		0.047
I	1 (reference)		1 (reference)	
II	2.633 (0.884-7.838)	0.082	1.569 (0.287-8.570)	0.603
III	2.619 (1.056-6.498)	0.038	4.596 (1.493-14.151)	0.008
IV	51.627 (5.465-487.700)	0.001	0.000 (0.000-NA)	0.988

*, univariate Cox regression analysis; HR, hazard ratio; CI, confidence interval; Other types, papillary, chromophobe and undifferentiated; NA, not applicable.

**Table 3:** Results of multivariate analysis of overall survival and disease-free survival in 139 patients with renal cell carcinoma.

Prognostic factor	Overall survival		Disease-free survival	
	HR (95% CI)	P*	HR (95% CI)	P*
CYP4A11 expression	1.114 (1.026-1.210)	0.010	1.034 (0.923-1.158)	0.561
PPARα expression	0.978 (0.882-1.084)	0.671	0.930 (0.813-1.064)	0.291
Age at operation	1.035 (0.999-1.072)	0.054	1.000 (0.962-1.039)	0.996
Sex				
Female	1 (reference)		1 (reference)	
Male	2.409 (0.805-7.211)	0.116	1.497 (0.479-4.684)	0.488
Pathologic stage		0.023		0.062
I	1 (reference)		1 (reference)	
II	3.003 (0.967-9.330)	0.057	1.652 (0.300-9.109)	0.564
III	2.555 (1.002-6.515)	0.049	4.424 (1.426-13.728)	0.010
IV	26.037 (2.612-259.542)	0.005	0.000 (0.000-NA)	0.987

*, multivariate Cox regression analysis; HR, hazard ratio; CI, confidence interval; Other types, papillary, chromophobe and undifferentiated; NA, not applicable.

## Abbreviations

RCC: Renal cell carcinoma
ccRCC: clear-cell renal cell carcinoma
pRCC: papillary renal cell carcinoma
chRCC: chromophobe renal cell carcinoma
CYP4: cytochrome P-450 4
20-HETE: 20-hydroxyeicosatetraenoic acid
PPARα: peroxisome proliferator-activated receptor-α
IHC: immunohistochemistry
RT-ddPCR: reverse transcription digital droplet polymerase chain reaction
FFPE: formalin-fixed paraffin-embedded
DFS: Disease-free survival
OS: Overall survival
H&E: hematoxylin and eosin
TNM: The tumor, node, and metastasis
AJCC: the American Joint Committee on Cancer
TMA: Tissue microarray
cDNA: complementary DNA
VHL: the von Hippel-Lindau gene
HIF: hypoxia-inducible factor
